# Dental Education—Rejoinder

**Published:** 1884-08

**Authors:** Jas. H. Harris

**Affiliations:** 271 N. Eutaw St.; Baltimore, Md.


### REPLY BY PROF. HARRIS.

In the July number of the INDEPENDENT PRACTITIONER appears an article on "Dental Education," signed by the faculty of the "Baltimore College of Dental Surgery," in reply to one upon the same subject written by Dr. B. M. Hopkinson, which appeared in the June number of the same journal. This article contains certain charges against me, the entire falsity of which I will endeavor to make fully apparent. To those who are acquainted with myself and certain members of the faculty of the Baltimore College, I feel that any reply from me is unnecessary, and while to those who are acquainted with either, the venom which characterizes the article must be sufficiently apparent; yet as I have nothing to conceal as to my conduct in the matter, I have concluded to answer briefly the salient points of the article. It is stated that Dr. C. W. S. Baldwin on February 19, 1884, called upon Dr. Gorgas, the Dean of the Dental Department of the University of Maryland, and not finding him at home was sent to me, and at our interview was so disgusted with my disparagement of others that he applied the next morning to the Dean of the "old Baltimore College" to matriculate. The true statement of what passed during the conversation between Dr. Baldwin and myself is as follows: The subject of graduation was soon introduced, and Dr. Baldwin seemed most anxious to graduate at the close of the current session. I informed him that this was utterly impossible, that our lectures, etc., were nearly over, and that to allow him to matriculate and come up for graduation at that late day in the session would be a violation of the rules of our department, and should he graduate, simply amount to selling him a diploma. He gave certain reasons why he could not present himself sooner, such as sickness, business, etc., all of which I told him we could not consider. I further said to him that if he simply wished to buy a diploma I felt satisfied that he could get one for a small sum of money. He then made inquiries of me respecting the Baltimore College of Dental Surgery, asking me if it did not stand well, etc.,; to this I replied, that in the estimation of some, perhaps it did, but in that of many others it did not. I leave it to any unprejudiced mind to determine if the real reason why Dr. Baldwin made no further attempts to see Dr. Gorgas, did not arise from the fact that I had informed him that it would be impossible for him to graduate at that session with us, and that, to obtain a diploma at the time he desired, he must seek it elsewhere. This was merely an affirmation of the statements of Dr. Gorgas in his letters to Dr. Baldwin, which are contained in the article in the July number.

The next charge, in reference to Drs. F. C. Barlow and C. A. Meeker, is put rather in the form of an innuendo than as a definite assertion. "They were then introduced to Dr. J. H. Harris, who under the circumstances broached the idea of their receiving an 'honorary degree' at the end of the session." What does this sentence mean? If any specific charge is intended, why is it not plainly and clearly stated? Is it the object of the writers to "darken wisdom with words," or do they wish to insinuate a charge and then leave a loophole of escape for themselves upon the proof of its falsity? If the charge is that I promised either of the gentlemen an honorary degree, I then most unhesitatingly pronounce it false. What I did say to Messrs. Meeker and Barlow was, that I would much prefer to see them receive an "honorary degree" rather than the regular degree at that late day in the session, as in the former case it would not be asserted that money was the moving power; but I also informed them that this was impossible with us, as we had determined at the formation of our department not to confer honorary degrees.

The last charge, which takes the shape of an affidavit, is not signed, nor is the city, county, or State where it was taken given, nor is the character of the judicial officer before whom this affidavit was taken disclosed, unless we are to infer from the letters "J. P." that he is a Justice of the Peace—located at some point unknown. Now I ask of my professional brethren if this is brave or manly to thus stab in the dark? Why is the name of the affiant withheld? Is it for a more suitable time? If so I am at a loss to conceive of a time more suitable than when the affidavit itself is published to the world, and while I most positively pronounce the statements contained therein to be a wilfully false distortion of any conversation which the affiant may have had with me, yet I do not propose to belittle myself by making any further reply until such time as the name of the person making the affidavit is disclosed, when I will be pleased to answer it fully and explicitly. In conclusion, I would add that as a piece of special pleading and in dodging the real issues, and raising those which are entirely foreign to the original charges of Dr. Hopkinson, the article would do credit to gentlemen of the legal fraternity. Yet one fact remains undisputed, that the gentlemen named could not have graduated at the Dental Department of the University of Maryland at its last session, and *did* graduate at the Baltimore College of Dental Surgery. One more word, when I will leave the subject for the present. I *never did guarantee* a diploma to *any man*, and am fully aware that I do not now, and never did constitute a whole faculty. Had such a guarantee been given to an applicant desiring to graduate upon so limited attendance upon lectures, his name would surely not appear with the graduates of the Baltimore Dental College. I am always fully satisfied when I feel that I have performed my individual duties, and the idea of my attempting to assume the responsibility of an entire faculty is simply nonsense.

JAS. H. HARRIS,

271 N. Eutaw St.

BALTIMORE, MD., July 11, 1884.

